# 

**Published:** 1890-02

**Authors:** 


					﻿N O T I C E.
All articles for publication, books for review, business communications, etc.,
must be sent to Editors, 1125 Spruce Street, Philadelphia.
Subscribers will please notice that this Journal will be sent until arrears
are paid and it is ordered discontinued.
INTERNATIONAL MEDICAL ANNUAL FOR 1890.
The eighth yearly issue of the International Medical Annual (for 1890) • is
announced for early delivery. The Prospectus gives a promise of excellencies
surpassing ail former editions. Its thirty-seven editors in the several depart-
ments are to give a summary of New Remedies alphabetically arranged, also
a r&umd of New Treatment in dictionary form; with reference to the medi-
cal literature of the world pertaining to the year’s progress of medicine.
Such a practical and helpful volume is of inestimable value to the medical
profession. In one volume of about 600 octavo pages; price, $2.75, post
free.
E. B. Treat, publisher, 5 Cooper Union, New York.
				

